# Newly innovated system to generate adjustable PEEP with a high-flow nasal cannula

**DOI:** 10.1186/s40635-024-00627-6

**Published:** 2024-04-27

**Authors:** Yu Onodera, Kenya Yarimizu, Tatsuya Hayasaka, Kaneyuki Kawamae, Masaki Nakane

**Affiliations:** 1 Advanced Critical Care Center, Yamagata University Hospital, Yamagata, Japan; 2 Department of Anesthesiology, Faculty of Medicine, Yamagata University, Yamagata, Japan; 3 Department of Anesthesia, Ohta-Nishinouchi Hospital, Fukushima, Japan

**To the Editor,**

A high-flow nasal cannula (HFNC) has become an essential respiratory support for patients with acute respiratory failure [1]. The physiologic effects of an HFNC include reduced dead space ventilation through the CO_2_ washout effect and generation of positive end-expiratory pressure (PEEP) [2]. Previous physiologic studies have shown that the PEEP produced by an HFNC is low and cannot be adjusted in a clinically relevant manner [2]. When patients with respiratory failure who are being managed with an HFNC require PEEP, the patients must be switched to continuous positive airway pressure (CPAP), non-invasive positive pressure ventilation, or invasive positive pressure ventilation [3] with loss of the ventilatory support of the HFNC generated by the CO_2_ washout effect.

Therefore, we devised a new system by merging a full-face mask and a PEEP valve with an HFNC (HFNC-P) and conducted a simulation-based experiment to determine the feasibility of further clinical experiments.

The experiment was conducted using a respiratory model consisting of a life-sized 3D-printed airway model connected to a Training and Test Lung ([TTL]; Michigan Instruments, USA). Breathing patterns were established as normal (compliance [C], 50 mL/cmH_2_O; resistance [R], 5 cmH_2_O/L/s; tidal volume [Vt], 500 mL; and respiratory rate [RR], 14/min), restrictive (C 20; R, 5; Vt, 300; and RR, 25), and obstructive (C, 80; R, 20; Vt, 700; and RR, 10). CO_2_ was infused into the TTL to achieve a P_ET_CO_2_ of 40 mmHg with each breathing pattern and without interface connected to the airway model.

With this respiratory model, the following interfaces were attached:

1. HFNC: HFNC ([Optiflow]; F&P, New Zealand) with flow rates of 20, 40, and 60 L/min.
2. CPAP mask: A full-face mask with a PEEP valve set to 5 or 10 cmH_2_O and a flow rate of 20, 40, and 60 L/min was introduced.
3. HFNC-P: HFNC combined with a full-face mask (Cough Ventec Japan, Inc., Japan) and a PEEP valve set to 5 or 10 cmH_2_O (Fig. 1).

**Fig. 1:**
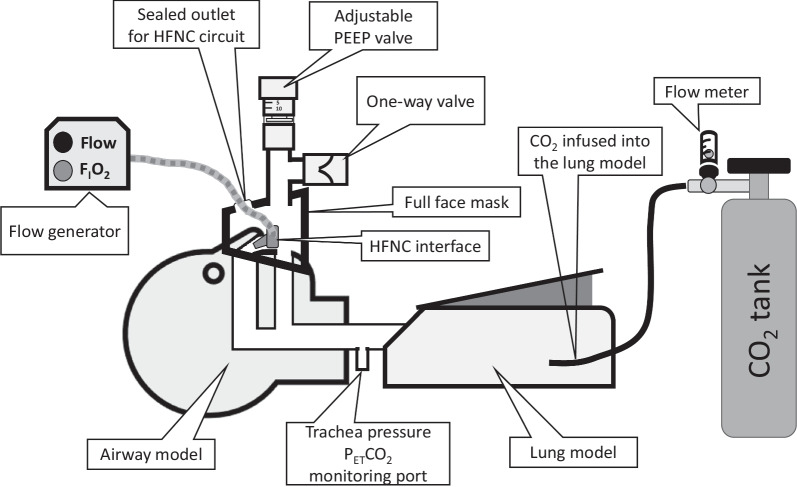
HFNC-P attached to the respiratory model. For HFNC only setting, the full-face mask was removed and for the CPAP setting, gas from the flow generator was directly infused into the full-face mask. A one-way valve was attached to the mask to accommodate external air inflow for CPAP and HFNC-P if the inspiratory flow surpassed the flow from the flow generator

PEEP and P_ET_CO_2_ in the trachea were measured for each setting.

As same as reported in our previous study, applying an HFNC was able to washout CO_2,_ reaching its maximum effect with a flow of 20 L/min, while PEEP only achieved 4 cmH_2_O with a flow of 60 L/min [2]. With the CPAP mask, P_ET_CO_2_ was reduced less compared to an HFNC, while achieving a PEEP level close to the PEEP valve setting with a flow setting > 40 L/min under normal and restrictive conditions and 60 L/min under obstructive conditions. By applying an HFNC-P, the washout effect was as effective as HFNC and able to produce PEEP close to the PEEP valve setting with a flow setting > 40 L/min (Fig. 2).

**Fig. 2:**
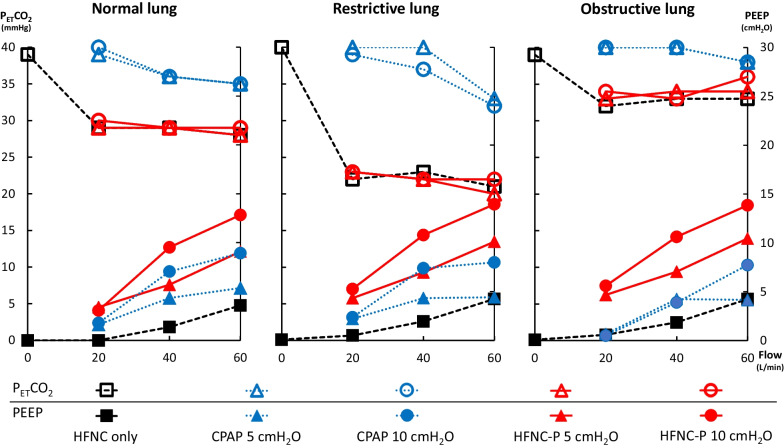
P_ET_CO_2_ and PEEP measured in each setting. Empty markers represent P_ET_CO_2_ data and filled markers represent PEEP data. HFNC-P was able to reduce P_ET_CO_2_ as much as HFNC and generate PEEP as much as CPAP

## Conclusion

Our newly innovated HFNC-P combines the HFNC washout effect and adjustable PEEP, which may accelerate the HFNC potential for respiratory support. Because this experiment was simulation-based and did not include patient data, approval by the Medical Device Regulation Committee and clinical studies assessing benefits and risk (excess or lack of humidification, skin ulcers, comfort, and cost effectiveness) is warranted.

## Disclosures

This work was presented at the 2023 Critical Care Canada Forum [4]. Yamagata University and Cough Ventec Japan, Inc. jointly obtained a patent for the HFNC-P in Japan (patent number, 7406681).

## Data Availability

The datasets supporting the conclusions of this article are included within the article.
